# The impact of direct aperture optimization on plan quality and efficiency in complex head and neck IMRT

**DOI:** 10.1186/1748-717X-7-7

**Published:** 2012-01-23

**Authors:** Marcello Sabatino, Matthias Kretschmer, Klemens Zink, Florian Würschmidt

**Affiliations:** 1 Department of Radiation Therapy and Radiooncology, Radiologische Allianz Hamburg, Hamburg, Germany; 2 Institute of Radiation Protection and Medical Physics,Technische Hochschule Mittelhessen, Giessen, Germany

**Keywords:** head-and-neck, planning study, 2-step-IMRT, direct aperture optimization

## Abstract

**Background:**

Conventional step&shoot intensity modulated radio therapy (IMRT) approaches potentially lead to treatment plans with high numbers of segments and monitor units (MU) and, therefore, could be time consuming at the linear accelerator. Direct optimization methods are able to reduce the complexity without degrading the quality of the plan. The aim of this study is the evaluation of different IMRT approaches at standardized conditions for head and neck tumors.

**Method:**

For 27 patients with carcinomas in the head and neck region a planning study with a 2-step-IMRT system (KonRad), a direct optimization system (Panther DAO) and a mixture of both approaches (MasterPlan DSS) was created. In order to avoid different prescription doses for boost volumes a simple standardization was realized. The dose was downscaled to 50 Gy to the planning target volume (PTV) which included the primary tumor as well as the bilateral lymphatic drainage (cervical and supraclavicular). Dose restrictions for the organs at risk (OAR) were downscaled to this prescription from high dose concepts up to 72 Gy. Those limits were defined as planning objectives while reaching definable PTV coverage with a standardized field setup. The parameters were evaluated from the corresponding dose volume histogram (DVH). Special attention was paid to the efficiency of the method, measured by means of calculated MU and required segments. Statistical tests of significance were applied to quantify the differences between the evaluated systems.

**Results:**

PTV coverage for all systems in terms of V_90% _and V_95% _fell short of the requested 100% and 95%, respectively, but were still acceptable (range: 98.7% to 99.1% and 94.2% to 94.7%). Overall for OAR sparing and the burden of healthy tissue with low doses no technique was superior for all evaluated parameters. Differences were found for the number of segments where the direct optimization systems generated less segments. Lowest average numbers of MU were 308 by Panther DAO calculated for 2 Gy fractions. Based on these findings the treatment time at the linear accelerator is the lowest for Panther DAO.

**Conclusions:**

All IMRT approaches implemented in the different treatment planning systems (TPS) generated clinically acceptable and comparable plans. No superior system in terms of PTV coverage and OAR sparing was found. Major differences in efficiency of the method in terms of calculated MU and treatment times were found.

## Background

The exploration and evaluation of the clinical role of IMRT software from different vendors for complex treatment planning in head and neck tumors has been the subject of numerous studies [1-9]. These studies compared static step&shoot and sliding windows IMRT with dynamic rotational IMRT with regards to PTV coverage and sparing of OAR. Because of the complexity of treatment delivery, more attention was paid to the efficiency of the method to reach the desired dose distribution. The aim was to reduce the complexity without any concession to the quality of a plan. A problem which can occur is that treatment time takes much longer for traditional IMRT techniques as compared to conformal radiotherapy techniques. The number of MU and segments are also a matter of concern.

Particularly direct optimization systems achieved good results compared with the conventional 2-step IMRT (first step: calculation of fluence modulated distribution, second step: conversion into a deliverable sequence of segments) in the above mentioned aspects [3,5,9-11]. Furthermore, these technique might be favorable concerning radiation protection aspects for the patient due to reduced collimator head leakage and possibly reduced scattered radiation from the patient [12].

This study compares 2-step and directly optimized IMRT approaches. The planning study was carried out retrospective on 27 previously contoured and clinically treated head and neck cases. Plans were calculated at standardized planning conditions in terms of same linear accelerator, gantry angles and planning objectives for PTV coverage and OAR sparing.

## Methods

### Treatment planning systems

IMRT plans were calculated with Panther DAO version 4.71 (Concord, CA, USA). The optimization was done with the direct aperture optimization (Panther DAO) approach. For this purpose a leaf is selected randomly and a Gaussian distribution determines step length and direction of the leaf. Simultaneously, the weighting of the aperture is optimized. A stochastic fast-simulated annealing algorithm is minimizing the objective function and has two possible options to overcome local minima. In order to favor "tunneling", this algorithm uses iteration-step sizes derived from Gaussian distributions. At the beginning, large steps are possible, which will be reduced during the optimization process [11]. Furthermore with the progress of optimization, the step size of "running up" the objective function is changed. The probability of accepting large steps "uphill" is controlled by the dynamic parameters of the temperature [13]. Transferred to the stochastic optimization algorithm, large steps in the direction of the maximum are allowed to escape from local minima in the beginning of the process. The variation of step size is generated from a Cauchy distribution. First large step sizes are possible and then rapid changes in step size are allowed [14].

The user must specify the number of apertures to be optimized for each field. Optionally, a minimum number of MU are determined for an aperture, which may not be violated. These conditions are, in addition to the dose prescriptions, taken into account directly during optimization. During the optimization process, the progress is calculated with a Pencil Beam algorithm (PB) and visualized with an objective function value and a dose volume histogram (DVH). If the result is satisfactory, the final dose calculation is performed with a collapsed cone convolution (CCC).

In Oncentra MasterPlan version 3.3 (Nucletron BV, Veenendaal, Netherlands) a Direct Step and Shoot License (DSS) is available to generate fluence modulated fields with a direct optimization approach. A deterministic gradient algorithm minimizes the objective function. In order to find a good starting point for the direct optimization procedure, fluence profiles are calculated as for the 2-step approach within a user defined number of iterations. Until the predefined iterations are reached, the conversion is administered in segments. The sequencer produces approximately the number of segments previously defined by the user. These segments are then incorporated into the optimization process and are optimized directly in all subsequent iterations. From that point on only the leaf placement and weighting of the apertures is varied. The number of segments as well as the position of the jaws remains unchanged. For the segmentation process a minimal number of MU per segment and minimum field openings are to be set. The dose calculation during optimization is performed with a simplified algorithm. In the conversion step and for the final calculation a full CCC is used [15].

KonRad is a 2-step inverse planning tool from Siemens AG (Erlangen, Germany). All plans used for this study were created with version 2.2.23. The optimization is carried out with a Gradient-Newton method, which optimizes fluence distributions. A leaf sequencer translates the fluence distribution into segments. The number of produced segments depends mainly on two factors: the chosen number of steps of intensity levels and the usage of a median filter. If the intensity levels are set to a high value, the leaf sequencer has the possibility to approach the calculated distribution with more segments, which, in general, leads to a better approximation of the initial fluence. Another influence is the usage of a median filter which smoothes strong gradients within the intensity distribution. The impact depends on the dimension of the filter [6]. For dose calculation a PB algorithm is used.

### Patient population and dose prescription

The study enrolled 27 patients with carcinomas in the head and neck region. Depending on the location of the tumor, the PTV included the primary tumor of the oro-, hypo-, nasopharynx or larynx and the bilateral cervical and supraclavicular lymphatic drainage (mean target volume:1209 ± 281 cm^3^). In the immediate vicinity the relevant OAR are the spinal cord, the brain stem (depending on the tumor location and extent of PTV) and the parotid glands. A total dose of 50 Gy was delivered to the PTV including the lymphatic drainage area and boost irradiation to a maximum of 72 Gy could follow. This might result in a wide variation of boost contours (volume, position, prescribed dose), for which a large number of plans with different dose prescriptions would have to be created. In order to achieve comparability between the systems a simple standardization was applied. Plans for the low-dose target area were created and set to a fractionation of 2 Gy in 25 fractions. As the total dose was kept to 50 Gy (and not to 72 Gy as it would be in the curative approach) the maximum dose affecting the spinal cord, parotid glands and brainstem was reduced proportionally. Thus, the maximum dose to the spinal cord and brainstem was kept below 30 Gy, and that to the parotid glands below 19 Gy [16]. Healthy tissue is defined as outer contour of the patient subtracted by the PTV. This volume is limited in craniocaudal direction.

### Planning methodology

The standard field setup used for all plans consisted of seven static 6 MV photon fields with gantry angles of 0°, 52°, 104°, 156°, 204°, 256°, 308° with a dose rate of 300 MU/min. All plans were calculated for a clinical used Artiste Linac (Siemens AG, Erlangen, Germany) equipped with a 160 Leaf MLC. Further details and dosimetric characteristics were investigated by Tacke et al. [17]. The planner tried to keep the number of segments as low as possible while fulfilling the planning goals. The maximum segment number allowed for DSS plans were kept to a minimum while reaching the planning objectives. In order to minimize the number of segments for KonRad the intensity levels and used median filter were varied. Since the number of segments per beam direction is fixed at Panther DAO due to user definitions, the planner was allowed to apply split beams to provide additional degrees of freedom. This procedure is doubling the beam from one direction and therefore doubling the number of segments from this particular direction. The cut off for all systems per segment was set to 5 MU with a minimum field size of 4 cm^2^.

Different to the other systems, Panther DAO allowes fluence modulation with fixed jaws so the modulation is done by the leaves themselves.

Planning objectives were formulated to avoid differences in the optimization weighting factors and the way these are interpreted by the planning software. The primary goal was to treat the PTV with a minimum of 95% and a maximum of 107% of the prescribed dose [18], which should ideally lead to a median dose of 50 Gy. No dose normalization took place. Furthermore, the volume of the PTV which received 90% and 95% of the prescribed dose (V_90%_/V_95%_) should reach 100% respective 95% of the target dose. Secondary objectives were the above mentioned dose limits to the OAR, provided that the targets goals were met satisfactorily. Consistent support structures were created for all systems in order to steer the optimization and to avoid overdose outside the PTV. These structures will be integrated into the planning process, but no planning objectives, in terms of V_xGy _should be below a certain percentage, were defined for these volumes. Further attempts were made to achieve the planning specifications with as few segments as possible.

### Evaluation methods

The plans were compared and analyzed using DVH. For all systems, except KonRad, the data are analyzed in each program's own analysis tool. Since KonRad has no output for specific D_x% _- and V_yGy _-values, the CT scans, dose and volume structures were exported into MasterPlan, where the plans were further analyzed. No recalculation of dose took place.

Because of the fact that the dose calculation algorithms of the treatment planning systems (TPS) have difficulties and different approaches to model the build-up effect, the PTV was retracted 3 mm from the outline [19]. This modified PTV was used for further analysis. Maximum doses were included in the assessment by the parameter of V_107%_. The homogeneity index (*HI *= [D_2%_-D_98%_]/D_prescription_) reflects how steep the dose drop off in the PTV is. A smaller *HI *indicates a more homogenous dose distribution.

Maximum doses in serial OAR are reflected on D_2%_. The dose to the major salivary glands was recorded at the median dose. Low-dose exposure of healthy tissue was reported as the volume which receives 5 Gy (V_5 Gy_) and 10 Gy (V_10 Gy_). For all plans the treatment times were measured from the beginning of the first field until the end of the last segment. The efficiency of the IMRT method was derived from the calculated MU and required segments.

To quantify the differences of parameters between two systems a test of significance is required. Since the measurements were collected for each planning system for the same collective, a two-sided, paired student t-test was used. Statistical significance was defined for p-values < 0.05. A Kolmogorov-Smirnov-test was applied before, whereby the parameters were tested with regard to a normal distribution.

All results of this study are reported as averages of the entire patient cohort and the appropriate standard deviation.

## Results

### Dose-coverage for PTV

**Figure 1:**
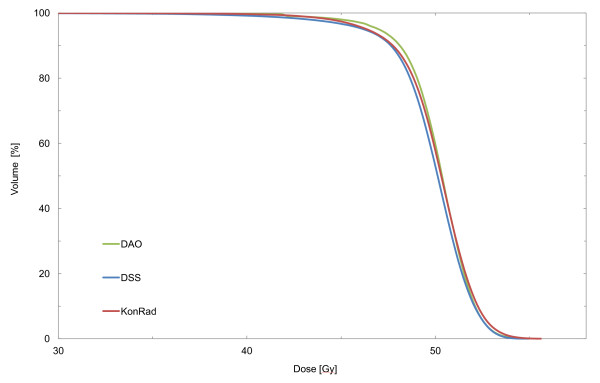
**Dose volume histogramm for the PTV for the three tested IMRT-systems**. Shown is the average DVH for the whole patient cohort, different colours denoting the different TPS.

All IMRT systems reach satisfactory and comparable results for the dose in the PTV. The prescribed median dose of 50 Gy is achieved in all cases. Figure 1 shows the average DVH for the entire patient cohort for the three TPS.

The prescribed high-dose objectives for V_90% _and V_95% _come close to the requested aim (100% respective 95%). The volume which receives more than 107% of the prescription dose is lowest for DSS (0.3%) followed by Panther DAO (0.7%) and KonRad (1.2%). Table 1 shows the results for the PTV according to the DVH analysis.

**Table 1:** Dosimetric results for the PTV from 2-step and direct optimized IMRT for the tested TPS.

Parameter	DAO	KonRad	DSS	p
Median [Gy]	50.3 ± 0.1	50.3 ± 0.1	50.3 ± 0.1	-
V_107% _[%]	0.7 ± 0.4	1.2 ± 0.6	0.3 ± 0.5	a, b, c
V_95% _[%]	94.7 ± 0.8	94.2 ± 1.3	94.7 ± 1.1	a
V_90% _[%]	99.1 ± 0.4	98.7 ± 1.1	99.1 ± 0.4	-
HI [-]	0.13 ± 0.01	0.14 ± 0.01	0.12 ± 0.01	a, b, c

Given is the median dose, the volume which receives 107%, 95% and 90% of the prescribed dose as well as the homogeneity index.

Statistical significance (p < 0,05): a: Panther DAO vs KonRad, b: Panther DAO vs DSS, c: KonRad vs DSS,

The *HI *is within a close range for all tested systems (0.12 for DSS; 0.13 for Panther DAO; 0.14 for KonRad) and has a low standard deviation (0.01). However, statistical significant differences for the *HI *were found for all planning systems. The p-values are < 0.001 for DSS vs. Panther DAO and KonRad, and Panther DAO vs. KonRad.

### Organs at risk and low dose exposure

The results of DVH analysis for the OAR are listed in Table 2. Only KonRad met the planning objectives for the serial riskstructures. The average maximum doses to the spinal cord are 30.0 Gy (KonRad), 30.6 Gy (DSS) and 31.5 Gy (Panther DAO). KonRad is best in sparing the brainstem with 26.8 Gy followed by DSS (27.2 Gy) and Panther DAO (30.9 Gy). Statistical significant differences are observed between Panther DAO and the two other TPS.

**Table 2:** Dosimetric results for spinal cord, brainstem, summed parotid glands and healthy tissue.

Organ	Parameter	DAO	KonRad	DSS	p
Spinal cord	D_2% _[Gy]	31.5 ± 1.1	30.0 ± 1.6	30.6 ± 1.4	a, b
Brainstem	D_2% _[Gy]	31.0 ± 2.2	26.8 ± 2.6	27.2 ± 2.6	a, b
Summed Parotids	D_Median _[Gy]	21.0 ± 1.8	21.3 ± 2.1	19.7 ± 1.8	b, c
Healthy Tissue	V_5 Gy _[%]	66.7 ± 5.4	73.5 ± 5.4	73.6 ± 4.2	a, b
Healthy Tissue	V_10 Gy _[%]	53.8 ± 5.8	57.8 ± 5.2	59.4 ± 3.8	a, b, c

Given is the dose in Gy to two percent of the volume of the spinal cord and brainstem, median dose to the parotid glands and the volume of healthy tissue receiving more than 5 Gy respective 10 Gy.

Statistical significance (p < 0,05); a: Panther DAO vs KonRad, b: Panther DAO vs DSS, c: KonRad vs DSS

The planning objective for major parotid gland sparing was difficult to achieve. The median dose varied between 19.7 Gy (DSS) to 21.0 Gy (Panther DAO) and 21.3 Gy (KonRad) (see Table 2).

The exposure of healthy tissue to doses below 5 Gy and 10 Gy is presented in Table 2. Statistical significance are observed for all values and planning systems except for V_5 Gy _between KonRad and DSS. The exposure at this dose level is lowest for Panther DAO followed by KonRad and DSS.

### Evaluation of efficiency

**Figure 2:**
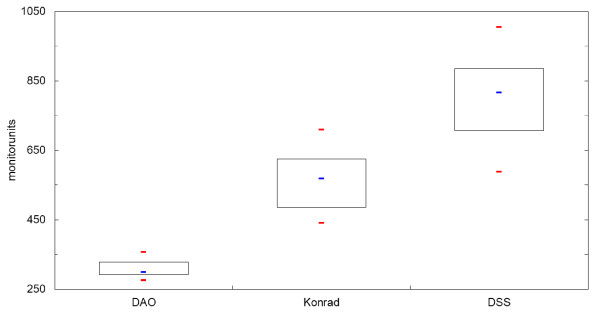
**Boxplot diagram of monitorunit distribution calculated from the three tested IMRT-systems**. The median is shown as a blue line, maximum and minimum in red, and 1^st ^and 3^rd ^quartile as thin black lines.

**Figure 3:**
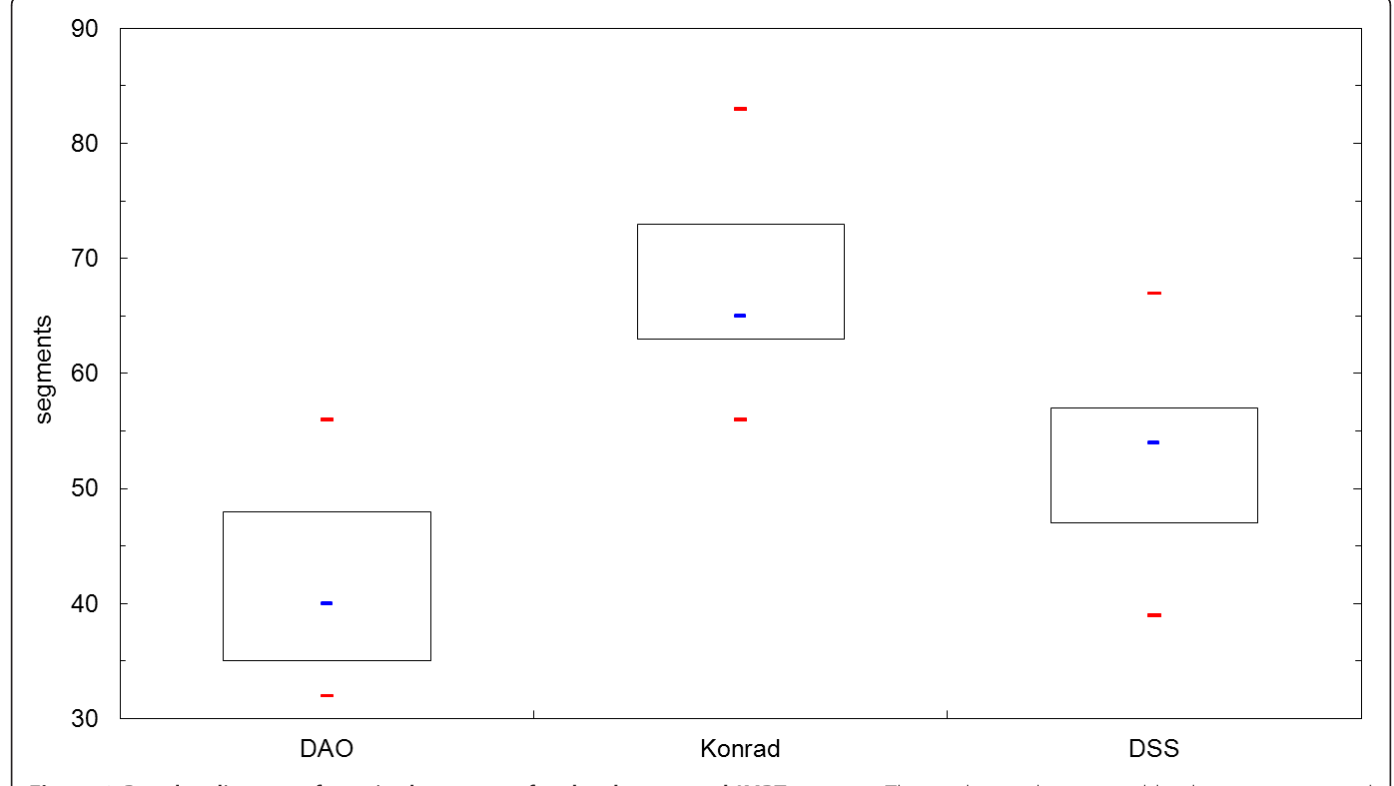
**Boxplot diagram of required segments for the three tested IMRT-systems**. The median is shown as a blue line, maximum and minimum in red, and 1^st ^and 3^rd ^quartile as thin black lines.

The number of MU for a 2 Gy fraction resulted in 308 ± 21 MU for Panther DAO, 564 ± 78 MU for KonRad and 807 ± 101 MU for DSS. Compared to DSS the percentages of MU reduction are 30% (KonRad) and 62% (Panther DAO). The results are shown in table 3 and as boxplot diagrams for the obtained MU in Figure 2. In a similar way the required segments are shown in Figure 3. On average 43 ± 9 segments are needed for Panther DAO, 53 ± 8 segments for DSS and 68 ± 7 segments for KonRad.

**Table 3:** Average MU and treatment time for the three different optimization systems.

Parameter	DSS	KonRad	DAO
MU	807 ± 110	564 ± 78	308 ± 21
reduction	-	30%	62%
treatment time [min]	10.5 ± 1.2	9.75 ± 1.2	7.0 ± 0.9
reduction [min]	-	0.75	3.5

Given are the calculated average MU for a 2 Gy fraction, the average treatment time and the respective reduction to the highest values (DSS).

Treatment times were measured for all patients and planning systems. The longest average treatment time was 10.5 ± 1.2 min for DSS. For the KonRad system 9.75 ± 1.2 min were observed and 7.0 ± 0.9 min for Panther DAO.

**Figure 4:**
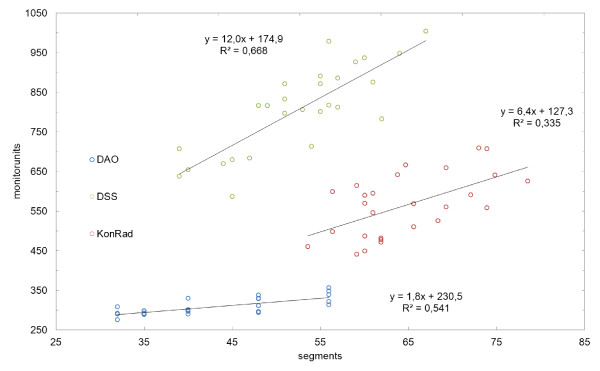
**Linear correlation between number of segments and MU**. The MU are given according to the number of segments for each TPS. The quality of the correlation is given with R^2^. Lines indicate fitted linear correlation and the corresponding equation is given.

Figure 4 shows the correlation between the MU and the number of segments. For DSS, the relationship between these variables is most pronounced (R^2 ^= 0,668). A moderate increase is recorded with the KonRad (R^2 ^= 0.335) system. Theoretically 80 segments with Panther DAO (R^2 ^= 0.541) would not exceed 400 MU.

## Discussion

This study compares step&shoot IMRT for head and neck tumors at standardized conditions with special attention to their different optimization approaches.

In terms of PTV coverage and OAR sparing all systems reach satisfactory and clinically acceptable results, even though some statistical significant differences can be observed. But the clinical relevance at this level is questionable. Similar conclusions could be stated for the risk structures spinal cord, brainstem and parotid glands. Panther DAO is violating the planning objectives but if one projects this percentage dose reduction to a total prescription dose of 72 Gy, sparing of the spinal cord is possible. Greater variations were observed in the efficiency of the intensity modulation. Compared to the 2-step approach the direct optimization algorithms are able to decrease the number of segments. DSS generates 22% and Panther DAO 37% less segments than KonRad. One aim was to keep the number of segments per plan as low as possible while reaching the planning goals for PTV coverage and OAR sparing. One reason for the efficiency of Panther DAO is the possibility to create a highly modulated field with few segments. With 5 segments per beam, 31 intensity levels (intensity levels = 2^n^-1) can be generated [11]. Concerning calculated MU for a 2 Gy fraction the purely direct optimization system has the lowest values. The reduction for Panther DAO is 62% and for KonRad 30% compared to DSS.

Statistical differences were found for the low dose exposure. Two reasons could be responsible for this finding: chosen gantry angles and dose calculation algorithms. In this case the large deviations could not be explained by the gantry angles since they were all the same for all plans and systems.

The presumption is that the differences occur due to less MU. But since the TPS calculations accuracy for low doses is reduced, an experimental measurement could verify these findings.

For Panther DAO - even if highly rising numbers of segments occurred - only a moderate increase of MU (R^2 ^= 0.541) can be expected. Rather a further increase of segments could result in potentially dosimetric unstable conditions, as the number of MU per segment may be too small. A stronger correlation was found for DSS (R^2 ^= 0.669). A larger variation of the pair of values occur in KonRad (R^2 ^= 0.335).

The primary focus of this study is the MU efficiency of the compared optimization algorithm. This is owed to the increasing number of MU in IMRT in comparison to 3D-conformal radio therapy (3DCRT) which could increase the risk of radiation induced secondary malignancies due to scattered radiation. Panther DAO plans could decrease the amount of scatter radiation originating from the collimator head. Hall pointed out the need for protection of patients from scattered radiation in IMRT-treatments [12]. He reported a potential increase of radiation-induced cancer due to larger total-body doses caused by leakage radiation. Considering this aspect, MU reduced plans with comparable quality should be preferred, especially for pediatric cases or diseases of young adults and adolescents with highly curable concepts.

The reported reductions are in agreement with published studies. Jones et al. compared 2-step IMRT with directly optimized IMRT plans using the Pinnacle DMPO in a planning study for head and neck tumors [5]. For this system an approach for the direct optimization, which is similar with DSS, is implemented. It is reported that DMPO requires 42% less MU and 35% less segments. The exposure time is reduced by 29%. Dobler et al. evaluated the effects of DSS compared to the 2-step approach of MasterPlan [3]. The study was conducted with 10 patients with a hypopharyngeal carcinoma with the same field arrangement and fraction dose as in this study. A reduction in MU from 1151 to 901 was found in favor of the direct optimization procedure. The required average segment number of 77 is the same for both approaches. In a further planning study concerning head and neck tumors with integrated boost, Wiezorek et al. compared static and rotational IMRT and Tomotherapy as well as different optimization algorithms [9]. Normalized MU were found to be lowest for Panther DAO.

The low number of MU and segments is the main reason for the shortest treatment times for Panther DAO. On average, the amount of time saved is 3.5 min (DSS) and 2.8 min (KonRad). These time savings could be used for image guidance. In addition to that it is advantageous for intrafractional movement of the organs and for the comfort of the patient. A reason for these time savings is the above mentioned creation of fluence modulation. Within a field the modulation is done by the fast leaves while the slower jaws are fixed to one position.

In the planning study by Wiezoreck et. al the exposure of healthy tissue to low doses were also evaluated. The low doses to healthy tissue were found to be highest for the Panther DAO system [9]. These findings differ from the results of our study. Differences may occur due to different approaches to calculate the values for the low dose exposure. In this study the external was subtracted by the PTV and the extent of this new volume limited to 3 cm in craniocaudal direction from the PTV. This was done because of a limited calculation matrix in KonRad.

Another reason could be the number of chosen gantry angles. In the study of Wiezoreck et al. eleven beam directions were taken for Panther DAO.

## Conclusions

All IMRT systems are able to calculate acceptable plans in terms of PTV coverage and OAR sparing. Main differences are observed in the efficiency of the fluence modulation. Based on the results of published literature and the results of this study, a further reduction of plan complexity can be stated for the purely direct-optimizing Panther DAO system in IMRT planning of complex head and neck cases. The reduced number of segments and MU should lead to less leakage radiation from the collimator head. If and how much these reductions lead to less peripheral doses should be verified by experimental measurements as performed by Wiezorek et al. [20].

## Competing interests

The Department of Radiation Therapy and Radiooncology of the Radiologische Allianz Hamburg receives research grants from Prowess Inc., Siemens AG Healthcare sector and Nucletron BV.

## Authors' contributions

MS and MK contributed significantly to study design and concept. MS was responsible for treatment planning. Data analysis and interpretation of results were performed by MS and MK. Corrections and/or improvements were suggested by MK, KZ and FW. FW was responsible for clinical evaluation of the treatment plans. All authors read and approved the final manuscript.
